# Xinnaotongluo liquid protects H9c2 cells from H/R-induced damage by regulating MDM2/STEAP3

**DOI:** 10.1371/journal.pone.0302407

**Published:** 2024-04-19

**Authors:** Jiankun Cui, Qinwen Wang, Minghao Li

**Affiliations:** 1 Department of Cardiology, The First Affiliated Hospital of Heilongjiang University of Chinese Medicine, Harbin, 150040, Heilongjiang, P. R. China; 2 Out-Patient Department, Beijing Garrison District Haidian Retired Cadres Twenty-Sixth, Beijing Garrison District Haidian Retired Cadres Twenty-Sixth, Beijing, China; 3 Department of Cardiology, Beidahuang Group General Hospital, Harbin, 150088, Heilongjiang, P. R. China; Instituto Nacional de Cardiologia Ignacio Chavez, MEXICO

## Abstract

Xinnaotongluo liquid has been used to improve the clinical symptoms of patients with myocardial infarction. However, the molecular mechanism of Xinnaotongluo liquid is not completely understood. H9c2 cells exposed to hypoxia/reoxygenation (H/R) was used to simulate damage to cardiomyocytes in myocardial infarction in vitro. The biological indicators of H9c2 cells were measured by cell counting kit-8, enzyme linked immunoabsorbent assay, and western blot assay. In H/R-induced H9c2 cells, a markedly reduced murine double minute 2 (MDM2) was observed. However, the addition of Xinnaotongluo liquid increased MDM2 expression in H/R-induced H9c2 cells. And MDM2 overexpression strengthened the beneficial effects of Xinnaotongluo liquid on H9c2 cells from the perspective of alleviating oxidative damage, cellular inflammation, apoptosis and ferroptosis of H/R-induced H9c2 cells. Moreover, MDM2 overexpression reduced the protein expression of p53 and Six-Transmembrane Epithelial Antigen of Prostate 3 (STEAP3). Whereas, STEAP3 overexpression hindered the function of MDM2-overexpression in H/R-induced H9c2 cells. Our results insinuated that Xinnaotongluo liquid could protect H9c2 cells from H/R-induced damage by regulating MDM2/STEAP3, which provide a potential theoretical basis for further explaining the working mechanism of Xinnaotongluo liquid.

## Introduction

With the improvement of people’s living standard, cardiovascular disease has become a kind of disease threatening human health [1]. Studies have shown that the current incidence and mortality of cardiovascular diseases have exceeded malignant tumors, becoming the first incidence of disease [2]. More than 5 million people die of cardiovascular disease each year, and the age of onset is trending younger. Myocardial ischemia (MI) is a multi-gene and multi-target disease, and it is difficult to achieve the expected therapeutic effect by using a single target [3]. A wide range of molecular mechanisms and pathophysiological changes occur during myocardial ischemia, including oxygen free radical activation, cell matrix proliferation, angiogenesis and neuronal differentiation, and remodeling of myocardial cells in ischemic areas [4–6]. These may be the mechanisms of myocardial tissue self-protection triggered by ischemic stimulation.

The components of traditional Chinese medicine (TCM) prescription are complex, and their therapeutic effects are often achieved through the integration of multiple components and targets [7]. TCM prescription has the characteristics of good therapeutic effect, strong activity, and low side effects in the treatment of MI disease, and has broad application prospects [8,9]. Xinnaotongluo liquid is an effective prescription developed by Professor Li Yan, a famous TCM professor at the First Affiliated Hospital of Heilongjiang University of Traditional Chinese Medicine [10–12]. However, the specific targets and molecular mechanisms still need to be further explored.

Murine double minute 2 (MDM2) has been reported to exert a role in MI by modulating inflammation in a p53-dependent manner. A study from Toth et al. indicated that cardiomyocytes overexpressing MDM2 acquired resistance to hypoxia/reoxygenation-induced apoptosis [13]. It was reported that apoptosis rates of myocardial cells increased in heart failure rats, through suppression of MDM2 expression [14]. Moreover, MDM2 was downregulated and significantly enriched in the PI3K/ATK signaling pathway in MI samples [15]. Additionally, MDM2 has been discovered to exert an important role in atherosclerosis by regulating p53 activity [16]. It is reported that storax effectively protected cardiomyocytes against myocardial fibrosis and cardiac dysfunction by upregulating MDM2 expression [17]. Our preliminary study found that Xinnaotongluo liquid can upregulate the expression of MDM2, but further research is needed.

Ferroptosis is a new form of cell death, mainly related to circulating iron content, manifested as excessive iron mediated imbalance of intracellular redox reactions, accumulation of lipid peroxides, and production of reactive oxygen species [18,19]. The pathological and physiological processes of many diseases are closely related to ferroptosis. A growing number of studies revealed that ferroptosis plays an important role in MI [20,21]. However, whether Xinnaotongluo liquid plays a role by regulating ferroptosis of cardiomyocytes during MI remains unknown.

In this study, a series of biological experiments were performed to detect the relationship between Xinnaotongluo liquid and MDM2/Six-Transmembrane Epithelial Antigen of Prostate 3 (STEAP3).

## Materials and methods

### Cell culture and transfection

The H9c2 cells were grown in Dulbecco’s Modified Eagle medium (DMEM) supplemented with 10% fetal bovine serum (FBS). The medium was changed every three days, and the cells maintained in 37°C atmosphere with 5% CO_2_. H9c2 cells were exposed to hypoxic condition supplemented with 0.1% O_2_, 5% CO_2_ and 95% N_2_ at 37°C for 6 h and then subjected to reoxygenation under normoxic condition equilibrated with 95% air and 5% CO_2_ at 37°C for 12 h.

Xinnaotongluo liquid was purchased from the Xianyang Buchang Pharmaceutical Co., LTD (Xi’an, China) and dissolved in saline solution. MDM2 overexpression (MDM2-OE) vector and STEAP3 overexpression (STEAP3-OE) vector were synthesized by using pcDNA3.1 vector. Transfection was performed by using the Lipofectamine 2000 reagent (Invitrogen, USA).

### Western blotting

The Radio Immunoprecipitation Assay (RIPA) lysis buffer, which containing protease inhibitor (Solarbio, China), was used to extract the proteins. Thereafter, the sodium dodecyl sulfate-polyacrylamide gel electrophoresis (SDS-PAGE) was used to separate the protein, which then electro-transferred onto polyvinylidene fluoride (PVDF) membranes. Next, the membranes were incubated with the primary antibodies overnight at 4°C, followed by incubated with the secondary antibodies conjugated with HRP for 1h at room temperature. Finally, the membranes were visualized and analyzed by using enhanced chemilum inescence and Image J software. The primary antibodies used in this study were listed as below: MDM2 (1:500, abcam), IL-1β (1:1000, abcam), IL-6 (1:1000, abcam), TNF-α (1:1000, abcam), Bax (1:3000, abcam), Bcl-2 (1:1000, abcam), cleaved caspase-3 (1:1000, abcam), GPX4 (1:1000, abcam), SLC7A11 (1:1000, abcam), p53 (1:1000, abcam), STEAP3 (1:1000, abcam), β-actin (1:2000, abcam).

### Cell proliferation detection

The viability of H9c2 cells was performed by using Cell Counting Kit-8 (CCK-8, Beyotime, Shanghai, China) based on the supplier’s direction. H9c2 cells were suspended in culture medium and seeded into 96-well plates with a density of 3000 cells per well for 24 h. After indicated treatment, cells were cultured with CCK-8 solution (10 μL/well) for additional 2 h. The absorbance was analyzed by using a microplate reader at the 450 nm.

### Oxidative stress indicators detection

In brief, H9c2 cells were seeded in 6-well plates and cultured overnight. After hypoxia treatment as described above, culture supernatant from each dish were collected, and lactate dehydrogenase (LDH) activity in medium was detected using commercial assay kits and was shown as U/mL. The malondialdehyde (MDA) content and antioxidant enzyme activity were detected by using commercial assay kit. The content of MDA was shown as nmol/mg protein. The activities of superoxide dismutase (SOD) and glutathione (GSH) were shown as U/mg protein.

### Iron assay

The intracellular iron was measured by iron assay kit. Briefly, H9c2 cells in 6-well plates were washed and then placed on ice. The following steps were performed according to the instructions of the iron assay kit. The absorbance was measured at OD593nm and standard curve was performed by according to manufacturer’s instruction.

### Statistical analysis

The whole experimental data was performed by using GraphPad Prism 8.0 software and presented as the mean ± standard deviation (SD). One-way ANOVA, followed by Bonferroni’s post hoc test, was applied for multiple comparisons. Differences with p value < 0.05 were considered to be significant.

## Results

### MDM2 overexpression strengthened the protective effects of Xinnaotongluo liquid on H/R-induced H9c2 cells

**Fig 1:**
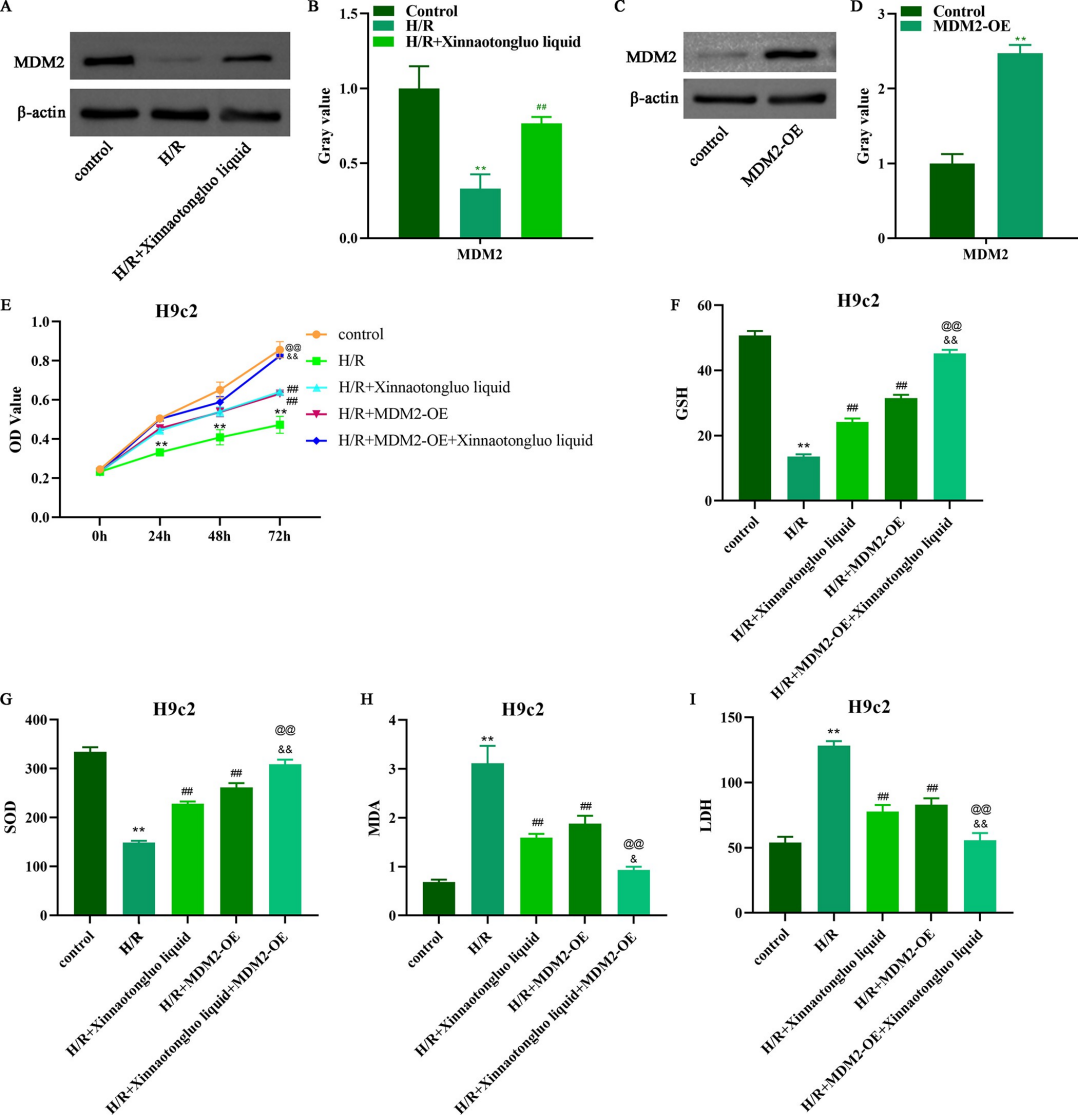
MDM2 was downregulated in H/R-induced H9c2 cells, and MDM2 overexpression helped Xinnaotongluo liquid to alleviate oxidative damage of H/R-induced H9c2 cells. A-B. Western blot was used to detect MDM2 expression in H/R-induced H9c2 cells after Xinnaotongluo liquid treatment. C-D. Western blot was used to detect MDM2 expression in H9c2 cells after MDM2-OE transfection. E. CCK-8 assay was used to detect the viability of H9c2 cells after Xinnaotongluo liquid and/or MDM2-OE treatment. F-I. The levels of GSH, SOD, MDA, and LDH were detected by corresponding commercial assay kits after Xinnaotongluo liquid and/or MDM2-OE treatment. **p<0.01 vs. control, ##p<0.01 vs. H/R, &&p<0.01 vs. H/R+Xinnaotongluo liquid, @@p<0.01 vs. H/R+MDM2-OE.

Under H/R stimulation, a significant reduction of MDM2 expression was occurred in H9c2 cells, whereas the addition of Xinnaotongluo liquid increased the expression of MDM2 (Fig 1A and 1B). To further detect the function of MDM2 in H/R-induced H9c2 cells, we artificially overexpressed MDM2 in H9c2 cells (Fig 1C and 1D). Thereafter, a series of indicators have been detected by biological experiments. The viability of H/R-induced H9c2 cells could be increased by adding Xinnaotongluo liquid alone or overexpression of MDM2 alone, but the effect was more obvious after Xinnaotongluo liquid and MDM2-OE co-treatment (Fig 1E). The levels of GSH and SOD in H/R-induced H9c2 cells were also increased by adding Xinnaotongluo liquid alone or overexpression of MDM2 alone, but the effect was more obvious after Xinnaotongluo liquid and MDM2-OE co-treatment (Fig 1F and 1G). Whilst, the levels of MDA and LDH in H/R-induced H9c2 cells were decreased by adding Xinnaotongluo liquid alone or overexpression of MDM2 alone, but the effect was more obvious after Xinnaotongluo liquid and MDM2-OE co-treatment (Fig 1H and 1I).

### MDM2 overexpression improved the effects of Xinnaotongluo liquid on inflammation, apoptosis, and ferroptosis of H/R-induced H9c2 cells

**Fig 2:**
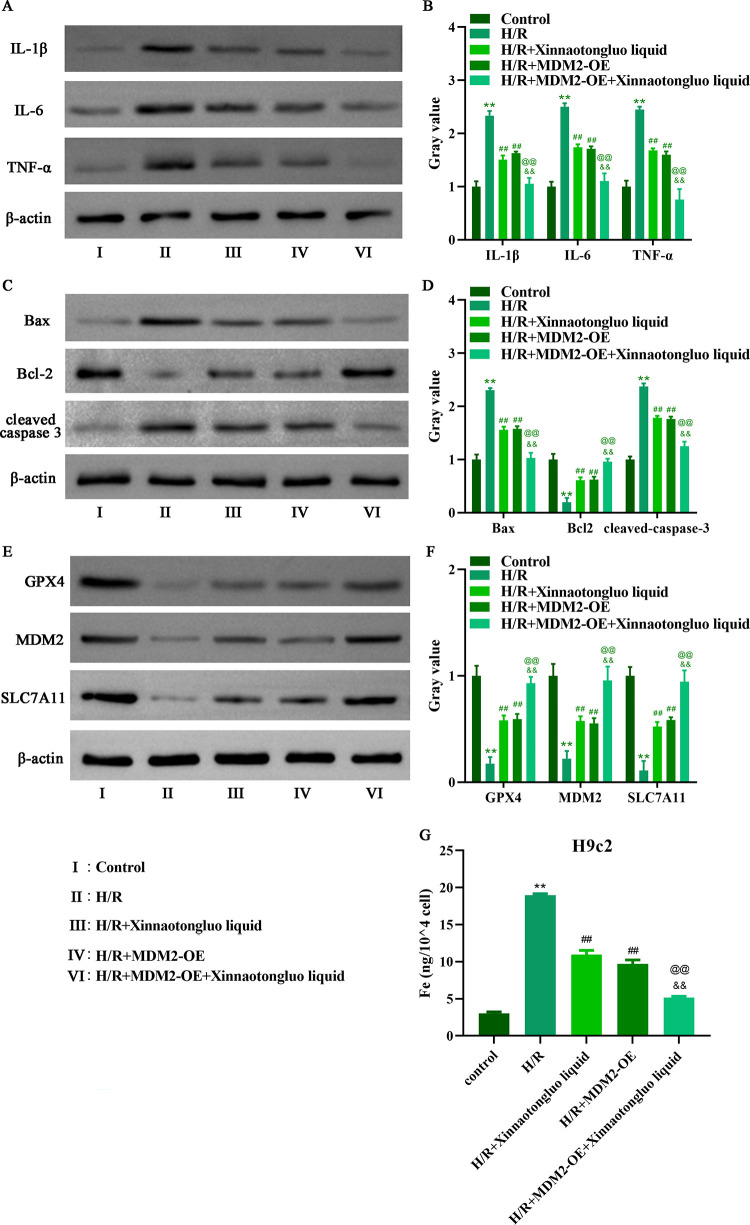
MDM2 overexpression contributed to the function of Xinnaotongluo liquid on the inflammation, apoptosis and ferroptosis of H/R-induced H9c2 cells. A-B. Western blot was used to detect IL-1β, IL-6 and TNF-α expression in H/R-induced H9c2 cells after Xinnaotongluo liquid and/or MDM2-OE treatment. C-D. Western blot was used to detect Bax, Bcl-2 and cleaved-caspase 3 expression in H/R-induced H9c2 cells after Xinnaotongluo liquid and/or MDM2-OE treatment. E-F. Western blot was used to detect GPX4, MDM2 and SLC7A11 expression in H/R-induced H9c2 cells after Xinnaotongluo liquid and/or MDM2-OE treatment. G. The iron level of H/R-induced H9c2 cells was measured after Xinnaotongluo liquid and/or MDM2-OE treatment. **p<0.01 vs. control, ##p<0.01 vs. H/R, &&p<0.01 vs. H/R+Xinnaotongluo liquid, @@p<0.01 vs. H/R+MDM2-OE.

Subsequently, western blot was performed to detect the protein expression of genes for inflammation, apoptosis, and ferroptosis. The protein levels of IL-1β, IL-6 and TNF-α were increased in H/R-induced H9c2 cells, whereas the addition of Xinnaotongluo liquid alone or overexpression of MDM2 alone reduced the protein levels of interleukin-1beta (IL-1β), interleukin-6 (IL-6) and tumor necrosis factor alpha (TNF-α) in H/R-induced H9c2 cells, which were more obvious after Xinnaotongluo liquid and MDM2-OE co-treatment (Fig 2A and 2B). The pro-apoptotic protein expression of Bax and cleaved caspase 3 were increased in H/R-induced H9c2 cells, whereas the addition of Xinnaotongluo liquid alone or overexpression of MDM2 alone reduced the protein levels of Bax and cleaved caspase 3 in H/R-induced H9c2 cells, which were more obvious after Xinnaotongluo liquid and MDM2-OE co-treatment (Fig 2C and 2D). However, the anti-apoptotic protein Bcl-2 showed the opposite effect when compared with Bax and cleaved caspase 3 (Fig 2C and 2D). The protein levels of glutathione peroxidase 4 (GPX4) and solute carrier family 7a member 11 (SLC7A11) were reduced in H/R-induced H9c2 cells, whereas the addition of Xinnaotongluo liquid alone or overexpression of MDM2 alone elevated the protein levels of GPX4 and SLC7A11 in H/R-induced H9c2 cells, which were more obvious after Xinnaotongluo liquid and MDM2-OE co-treatment (Fig 2E and 2F). Data from Fig 2G showed that the addition of Xinnaotongluo liquid alone or overexpression of MDM2 alone diminished the iron accumulation of H/R-induced H9c2 cells, which were more obvious after Xinnaotongluo liquid and MDM2-OE co-treatment.

### STEAP3 overexpression diminished the function of MDM2 in H/R-induced H9c2 cells

**Fig 3:**
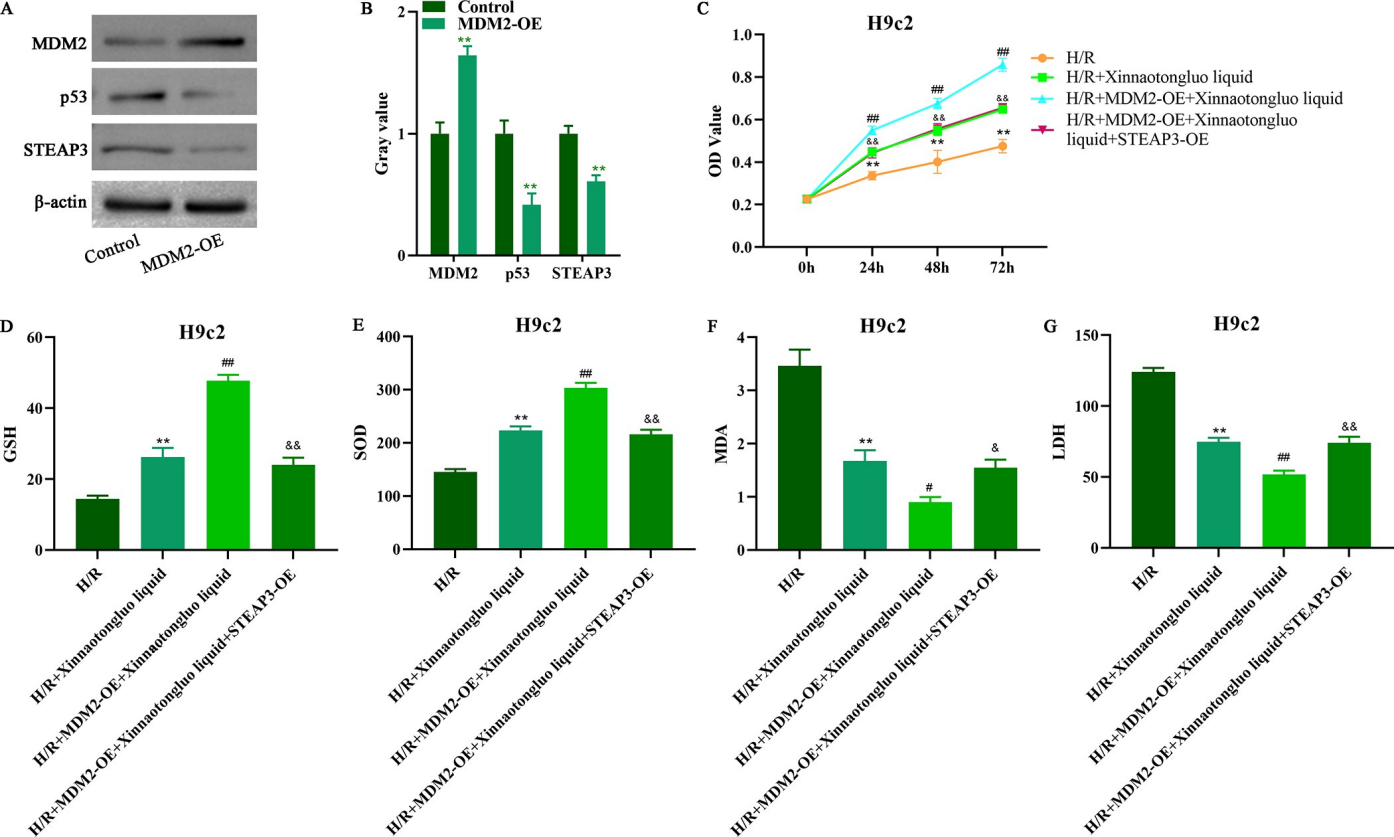
STEAP3 overexpression reversed the function of MDM2-OE on H/R-induced H9c2 cells. A-B. Western blot was used to detect MDM2, p53 and STEAP3 expression in H9c2 cells after MDM2-OE transfection. C. CCK-8 assay was used to detect the viability of H9c2 cells after Xinnaotongluo liquid, MDM2-OE and STEAP3-OE treatment. D-G. The levels of GSH, SOD, MDA, and LDH were detected by corresponding commercial assay kits after Xinnaotongluo liquid, MDM2-OE and STEAP3-OE treatment. **p<0.01 vs. control, ##p<0.01 vs. H/R+Xinnaotongluo liquid, &&p<0.01 vs. H/R+MDM2-OE+Xinnaotongluo liquid.

**Fig 4:**
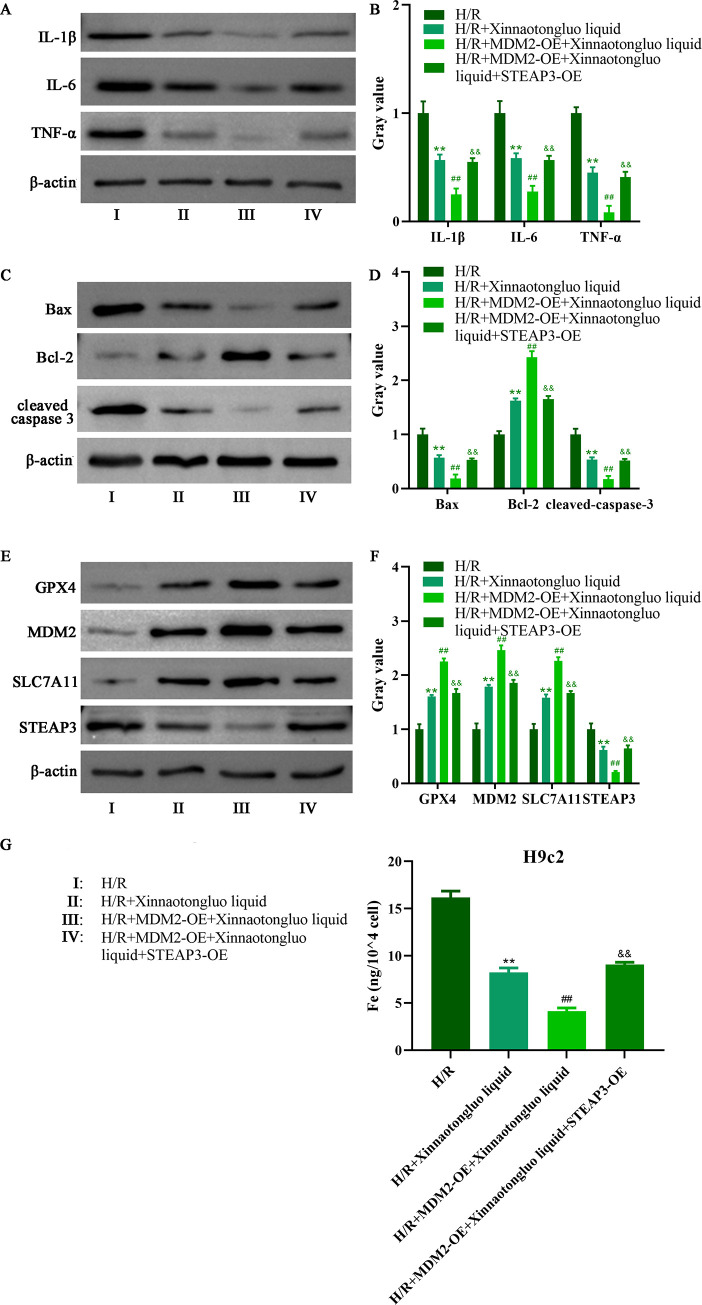
The function of MDM2-OE on H/R-induced H9c2 cells was hindered by STEAP3 overexpression. A-B. Western blot was used to detect IL-1β, IL-6 and TNF-α expression in H/R-induced H9c2 cells after Xinnaotongluo liquid and/or MDM2-OE treatment. C-D. Western blot was used to detect Bax, Bcl-2 and cleaved-caspase 3 expression in H/R-induced H9c2 cells after Xinnaotongluo liquid and/or MDM2-OE treatment. E-F. Western blot was used to detect GPX4, MDM2 and SLC7A11 expression in H/R-induced H9c2 cells after Xinnaotongluo liquid and/or MDM2-OE treatment. G. The iron level of H/R-induced H9c2 cells was measured after Xinnaotongluo liquid and/or MDM2-OE treatment. **p<0.01 vs. control, ##p<0.01 vs. H/R+Xinnaotongluo liquid, &&p<0.01 vs. H/R+MDM2-OE+Xinnaotongluo liquid.

Thereafter, we detected the expression of p53 and STEAP3 after MDM2 overexpression. Data from Fig 3A and 3B showed that MDM2 upregulation significantly reduced the protein levels of p53 and STEAP3. Then, we artificially overexpressed MDM2 and STEAP3 in H/R-induced H9c2 cells. We discovered that STEAP3 overexpression reduced the beneficial effects of MDM2-OE on H9c2 cells viability, and the opposite change occurred in GSH, SOD, MDA and LDH levels after STEAP3 overexpression (Fig 3C–3G). Similarly, the decline in IL-1β, IL-6 and TNF-α expression caused by MDM2-OE was reversed via STEAP3 overexpression (Fig 4A and 4B). Moreover, the reduction in Bax and cleaved caspase 3 expression, as well as the increase in Bcl-2 expression induced by MDM2-OE presented opposite changes after STEAP3 overexpression (Fig 4C and 4D). The increase in GPX4 and SLC7A11 expression induced by MDM2-OE was reversed via STEAP3 overexpression (Fig 4E and 4F). The reduction of iron level caused by MDM2-OE was inhibited after STEAP3 overexpressed (Fig 4G).

## Discussion

MDM2 is an intracellular protein with multiple biological functions. It was originally described as limiting p53-mediated cell cycle arrest and is considered a potential therapeutic target for cancer therapy [22]. However, MDM2 has many roles that are not related to p53. Several recent reviews have comprehensively discussed the function of MDM2 in tumorigenesis, its amplification and expression in diverse human cancers, and the cancer therapeutic agent that specifically target MDM2 protein [23–25]. Although the role of MDM2 in malignancy and tumor progression has been the focus of most previous studies, MDM2 has recently received increasing attention for its non-carcinogenic effects in various diseases and conditions, such as cardiovascular disease [26]. A previous study indicated that a markedly reduced MDM2 mRNA levels was observed in the hearts of wild type mice subjected to MI or trans-aortic banding [26]. In this study, we observed that MDM2 was decreased in H9c2 cells after H/R treatment, whereas Xinnaotongluo liqud treatment reversed this phenomenon. Moreover, in H/R-induced H9c2 cells, overexpression of MDM2 increased the levels of GSH and SOD, decreased the levels of MDA and LDH, reduced the protein levels pro-inflammatory and pro-apoptotic factors, as well as increased the protein levels of Bcl-2, GPX4 and SLC7A11.

Six-transmembrane epithelial antigen of the prostate 3 (STEAP3) is a member of the STEAP family and is essential for the absorption of iron and copper [27,28]. Additionally, STEAP3 null mice also presented their protective effects after ischemia-reperfusion injury by inhibiting transforming growth factor-β-activated kinase activation [29]. Cells overexpression STEAP3 were more sensitive to apoptosis [30]. In our study, overexpression of MDM2 significantly reduced STEAP3 expression, whereas STEAP3 overexpression hindered the function of MDM2-OE in H/R-induced H9c2 cells.

Ferroptosis, a cell death process driven by cellular metabolism and iron-dependent lipid peroxidation, has been involved in multiple diseases [31–33]. Ferroptosis occurs not only in cancer cells, but also in neurons, even in cardiomyocytes, for example, with the progression of reperfusion injury triggered by revascularization after coronary artery occlusion, cardiomyocytes appear iron sag, releasing inflammatory mediators and aggravating heart injury [31]. In addition, multiple clinical studies have demonstrated that myocardial iron is a crucial independent risk factor for left ventricular remodeling after MI [20,34]. Iron apoptosis can be activated by iron overload or by the inactivation of GPX4, a major endogenous mechanism that prevents peroxidation [19,35]. Peroxidation converts potentially toxic lipid hydroperoxides into non-toxic lipid alcohols. In the latter case, iron deposition can be inhibited by activating GPX4 [19]. SLC7A11 and GPX4 are inhibitors of ferroptosis, and suppression of SLC7A11 and GPX4 by different methods is able to induce ferroptosis [36]. In this study, Xinnaotongluo liqiud promoted the expression of GPX4 and SLC7A11, and inhibited the level of iron by upregulating MDM2 expression, suggesting that Xinnaotongluo liqiud inhibited the ferroptosis of H9c2 cells by up-regulating MDM2.

### Conclusions

In summary, our results demonstrated that Xinnaotongluo liquid could protect H9c2 cells from H/R-induced damage by regulating MDM2/STEAP3, providing a potential theoretical basis for further explaining the working mechanism of Xinnaotongluo liquid.
